# Real-World Impact of Switching From Insulin Glargine (Lantus^®^) to Basaglar^®^ and Potential Cost Saving in a Large Public Healthcare System in Saudi Arabia

**DOI:** 10.3389/fpubh.2022.852721

**Published:** 2022-06-13

**Authors:** Yazed AlRuthia, Ohud H. Bahari, Suliman Alghnam, Ali M. Alrumaih, Hassan Asiri, Mohammed Alshammari, Mansour Alhowimel, Hana A. Al-Abdulkarim

**Affiliations:** ^1^Department of Clinical Pharmacy, College of Pharmacy, King Saud University, Riyadh, Saudi Arabia; ^2^Pharmacoeconomics Research Unit, Department of Clinical Pharmacy, College of Pharmacy, King Saud University, Riyadh, Saudi Arabia; ^3^Department of Pharmaceutical Care, King Saud Medical City, Riyadh, Saudi Arabia; ^4^King Abdullah International Medical Research Center (KAIMRC), King Saud Bin Abdulaziz University for Health Sciences (KSAU-HS), King Abdulaziz Medical City, National Guard Health Affairs, Riyadh, Saudi Arabia; ^5^Pharmaceutical Care Department, Medical Services for Armed Forces, Ministry of Defense, Riyadh, Saudi Arabia; ^6^Drug Policy and Economic Center, National Guard Health Affairs, Riyadh, Saudi Arabia; ^7^National Unified Procurement Company (NUPCO), Riyadh, Saudi Arabia

**Keywords:** diabetes mellitus, insulin glargine, Lantus^®^, Basaglar^®^, cost savings, Saudi Arabia

## Abstract

**Background:**

The advent of Basaglar^®^, which is a biosimilar insulin glargine formulation for Lantus^®^ has brought hope that it will result in similar outcomes and lower costs. However, some health practitioners raised some concerns about the therapeutic equivalence of this new biosimilar. Therefore, we aimed to examine the clinical and financial impact of switching from Lantus^®^ to Basaglar^®^.

**Methods:**

This was a single–center retrospective chart review study of adult patients (e.g., ≥18 years) with diabetes mellitus (DM) who were treated with insulin glargine (Lantus^®^) for at least 12 months and then switched to Basaglar^®^ for another 12 months. The potential cost savings for the years 2018 to 2021 and the cost avoidance for 2022 were estimated using different conversion ratios between the two insulin glargine products (Basaglar^®^ and Lantus^®^) and acquisition prices.

**Results:**

One–hundred patients with DM who were previously treated with Lantus^®^ and switched to Basaglar^®^ were retrospectively recruited. About two–thirds of the patients (68%) had type 2 DM, and the male and female patients were equally represented. The mean glycated hemoglobin (A1C) at baseline was 9, and the mean difference in the A1C levels before and after switching to Basaglar^®^ was not significant (0.18, *p*-value = 0.503, 95% CI [−0.36–0.72]). Although the difference in the total daily insulin units between Lantus^®^ and Basaglar^®^ was not significant, the difference was leaning toward statistical significance despite the small sample size (−1.88, *P*-value = 0.25, 95% CI [−5.15–1.38]). Switching from Lantus^®^ to Basaglar^®^ could have led to significant cost savings that would range from approximately 1.77 to 23.7 million United States Dollars (USD) for the years 2018 to 2021 assuming an equal conversion ratio. However, those cost savings might not be realized if the switching to Basaglar^®^ required higher daily insulin units, and the difference in the public tender acquisition price between Lantus^®^ and Basaglar^®^ is less than 15%.

**Conclusion:**

Basaglar^®^ and potentially other biosimilar insulin glargine products can lead to significant cost savings without compromising the quality of care. However, their acquisition prices should be discounted.

## Introduction

Diabetes mellitus (DM) is a prevalent chronic health condition with serious complications if left untreated, such as stroke, chronic kidney disease, and myocardial infarction (1). DM is characterized by an elevation in the blood glucose levels that require close monitoring and effective control (2). It is believed that more than 476 million people worldwide suffer from either type 1 or type 2 DM, and this number is expected to rise to 570.9 million by 2025 (1). Moreover, the current global incidence of DM is approximately 23 million people annually (1). In Saudi Arabia, the prevalence and incidence of DM are one of the highest in the world (3). It is believed that around 7 million people suffer from DM and 3 million have prediabetes in Saudi Arabia (3, 4).

Although multiple oral and injectable medications have been approved for the management of DM, insulin remains the mainstay therapy, especially for type 1 DM (5, 6). Different insulin types exist today for different indications, such as rapid–acting, short–acting, intermediate–acting, and insulin mixtures. Short–acting regular insulin is administered subcutaneously usually 30 min before the meal with a duration of 5 to 8 h (7, 8). Similarly, the rapid–acting insulin, such as lispro and aspart, are administered subcutaneously before meals, however, they should be administered 15 min before meals due to their faster onset in comparison to regular insulin (8). Moreover, they are associated with a lower incidence of postprandial hypoglycemia and better control of postprandial glucose levels (8). On the other hand, intermediate–acting insulin, such as the Neutral Protamine Hagedorn (NPH), takes approximately 2 h to work and lasts 10–16 h (9). Therefore, it is available in different mixtures with regular and rapid–acting insulins [e.g., NPH/regular (70/30), protamine/lispro (50/50), and protamine/aspart (70/30)] to make its onset of action faster (9). Basal insulins, such as insulin glargine, insulin detemir, and insulin degludec are widely used in the management of both type 1 and type 2 DM (10–13). Their onsets and durations of action are variable and range from 1 to 4 h and 20 to 42 h, respectively (12). Although basal insulins, such as degludec and glargine, were found to have lower rates of hypoglycemic events in comparison to NPH insulin, no significant differences were found between them with regard to common side effects, such as weight gain and local site reactions (10, 14). However, insulin degludec appears to show lower rates of hypoglycemic events in comparison to insulin glargine among patients with type 2 diabetes but not type 1 (14). Nonetheless, insulin glargine has by far the largest market share in comparison to other basal insulins (12, 15).

Insulin prices have witnessed a dramatic increase over the past decade putting a huge strain on the budgets of different healthcare systems (16–18). The approval of long–acting biosimilar insulins with comparable safety and efficacy profiles to that of originators has improved the prospects of better affordability and access to those highly essential therapeutic molecules (18, 19). In 2014, Basaglar^®^ (insulin glargine) has been launched by Eli Lilly (Eli Lilly and Company, Indianapolis, IN, USA) and Boehringer Ingelheim (Ingelheim am Rhein, Germany) as the first follow–on biologic or biosimilar insulin to Sanofi's Lantus^®^ (Sanofi Aventis, Paris, France) and was approved by the European Union and tentatively by United States Food and Drug Administration (USFDA) (20, 21). Shortly after that, it was approved by the USFDA in December 2015 (22, 23). Basaglar^®^ has shown comparable safety and efficacy profiles to Lantus^®^ among patients with type 1 and type 2 DM based on two randomized controlled trials (ELEMENT-1 and ELEMENT-2) (24, 25). However, these studies did not provide sufficient evidence to support the interchangeability or substitution between Lantus^®^ and Basaglar^®^ (26). Nonetheless, some observational studies have shown no difference in terms of a change in the glycated hemoglobin levels (A1C) or daily insulin units. Moreover, the switch from Lantus to Basaglar resulted in significant cost savings according to a recently published retrospective observational study that included 225 patients from five different clinics affiliated with three different healthcare systems in the State of Iowa, United States (27).

In Saudi Arabia, the utilization of insulin is one of the highest in the world due to the high prevalence of DM among the local population (3, 4). The most conservative estimate of the direct medical cost of diabetes based on the Saudi Ministry of Health (MoH) data is 17 billion Saudi Riyals (USD 4.53 billion) in 2014, and this figure is expected to rise to USD 7.2 billion if those with undiagnosed diabetes join the treatment pool (28). Therefore, different strategies to minimize the financial burden of DM on the public healthcare sector are explored, and switching to less expensive biosimilar insulins is one of them (19). However, several Saudi public healthcare sectors are reluctant to switch from Lantus^®^ to Basaglar^®^ mainly due to the perceptions among some physicians that Basaglar^®^ is not therapeutically equivalent to Lantus^®^ and may lead to poor glycemic control if patients were switched to it. Thus, we aimed to explore the impact of switching from Lantus^®^ to Basaglar^®^ on the A1C levels and budget of a large military health system in Saudi Arabia.

## Methods

### Study Design and Setting

This was a retrospective review of electronic medical records (EMRs) from the National Guard Health Affairs (NG–HA). The NG–HA is one of the largest public health systems in Saudi Arabia that was founded in 1982 and has two large medical cities and multiple hospitals and primary care clinics with more than 3,000–bed capacity. Data from different institutions across the kingdom that are affiliated with the NG–HA were reviewed. The study was approved by the ethics committee of the King Abdullah International Medical Research Center, Riyadh, Saudi Arabia (approval no. RC20/608/R). No consent forms were needed since the study only involved the review of EMRs, and no personal identifiers of patients (e.g., name, phone number, and national identification number) were collected. The study adhered to the ethical principles of the Helsinki declaration and all collected data were anonymized and kept confidential (29).

### Population

Adult patients (e.g., ≥18 years) with type 1 or type 2 DM who have been treated with insulin glargine (Lantus^®^) for at least 12 months before being switched to Basaglar^®^ and treated for at least another 12 months were included in the study. Patients who have not been treated with Lantus^®^ before Basaglar^®^, those who have not been treated with Lantus^®^ or Basaglar^®^ for at least 12 months, and those who have their treatment plans modified, such as an increase or discontinuation of medication for DM, were excluded. Furthermore, patients with missing observations, such as the A1C, were excluded. Additionally, those who have their DM medications changed during the first 12 months after the switch to Basaglar^®^ were excluded. Demographic patient characteristics, such as age and gender, weight, duration of illness in years, other chronic health conditions (e.g., hypertension, dyslipidemia, cardiovascular disease), glycated hemoglobin (A1C), daily units of rapid–acting insulin (e.g., aspart), and the number of other hypoglycemic medications were collected.

### Statistical Analysis

The patients' baseline characteristics were reported using descriptive statistics, such as mean standard deviation, frequencies, and percentages. Daily insulin units, A1C, and weight were compared before and after the switch to Basaglar^®^ using paired Student's *t*-test.

To examine the budget impact of replacing Lantus^®^ with Basaglar^®^, the total number of procured pre-filled pens of Lantus for the years 2018 to 2022 were retrieved from one of the largest military healthcare systems in Saudi Arabia, and the potential cost savings were estimated based on a scenario of total replacement of the procured number of pre–filled pens of Lantus^®^ with Basaglar^®^. This method was used due to the lack of accurate statistics (e.g., prevalence and incidence rates) about the number of patients with DM in these institutions. Therefore, examining the budget impact using the real procured quantities for the years 2018 to 2022 should represent the true budget impact. The acquisition prices for both Lantus^®^ and Basaglar^®^ for each unit (pre-filled pen) were retrieved from the Saudi Food and Drug Authority (SFDA) website, which shows the public prices of different registered medications (30). Moreover, sensitivity analyses were conducted by varying the acquisition prices for each unit of Lantus^®^ and Basaglar^®^ based on the opinions of five individuals working in the pharmaceutical planning and purchasing in five different public health sectors in Saudi Arabia. Additionally, the potential procured quantities of Basaglar^®^ were varied based on the 95% confidence limits of the mean difference in total daily insulin units between Lantus^®^ and Basaglar^®^. Statistical analyses were conducted using SAS^®^ version 9.4 (SAS Institute, Cary, NC, USA), and Microsoft^®^ Excel 2016.

## Results

Using the EMRs queries, data for 117 patients who were switched from Lantus^®^ to Basaglar^®^ were retrieved. However, 17 patients were excluded due to missing observations resulting in a sample of 100 patients. The female and male patients were equally represented in the sample and the patients' mean age was 54 years. Most of the patients (68%) had type 2 DM, and hypertension and cardiovascular disease were the most commonly encountered comorbidities (e.g., >50%). The mean duration of illness, number of comorbidities, weight, A1C, and number of oral antidiabetic medications are shown in Table 1. The mean difference in insulin glargine daily units was −1.88 units (95% CI: −5.1491–1.376) lower for Lantus^®^ in comparison to Basaglar^®^. With regard to insulin aspart, the mean daily units for Lantus^®^ was 1.93 units (95% CI: −6.22–10.08) higher than Basaglar^®^. The mean A1C was 0.18% (95% CI: −0.36–0.72) higher with Lantus^®^ in comparison to Basaglar^®^. The patients' mean weight was 4.19 kg (95% CI: 1.17–7.22) higher with Lantus^®^ than Basaglar^®^. However, these differences except for weight were not statistically significant as shown in Table 2.

**Table 1:** Patient baseline characteristics.

**Characteristics**	**Total (*N =* 100)**
**Age in years, mean** **±SD**	54.41 ± 21.91
**Gender**, ***N*** **(%)**	
Male	50(50%)
Female	50(50%)
**Type of diabetes**, ***N*** **(%)**	
Type 1 diabetes	32 (32)
Type 2 diabetes	68 (68)
**Duration of illness in years, mean** **±SD**	3.95 ± 0.53
**Comorbidities**, ***N*** **(%)**	
Dyslipidemia	38 (38)
Hypertension	52 (52)
Cardiovascular disease	56 (56)
**Number of comorbidities, mean** **±SD**	2.54 ± 1.97
**Weight in KG, mean** **±SD**	77.15± 20.83
**Number of oral antidiabetic medications, mean** **±SD**	0.522 ± 0.848
**Hemoglobin A1C, mean** **±SD**	9.05± 2.04

**Table 2:** Number of daily units for the two insulin glargine formulations (Lantus^®^ and Basaglar^®^) and insulin aspart and the difference between the A1C levels.

**Variable**	**Lantus^®^, Mean ±SD**	**Basaglar^®^,** **Mean ±SD**	**Difference (95% CI)**	***P*-value**
Insulin glargine daily units	28.23 ± 14.94	30.11 ± 15.44	−1.88(−5.1491–1.376)	0.250
Insulin aspart daily units	41.06 ± 24.83	39.14 ± 28.68	1.93(−6.22–10.08)	0.635
A1C	9.05 ± 2.04	8.86 ± 1.72	0.18(−0.36–0.72)	0.5032
Weight in KG	77.15± 20.83	72.95 ± 20.12	4.19(1.17–7.22)	0.007

**Figure 1:**
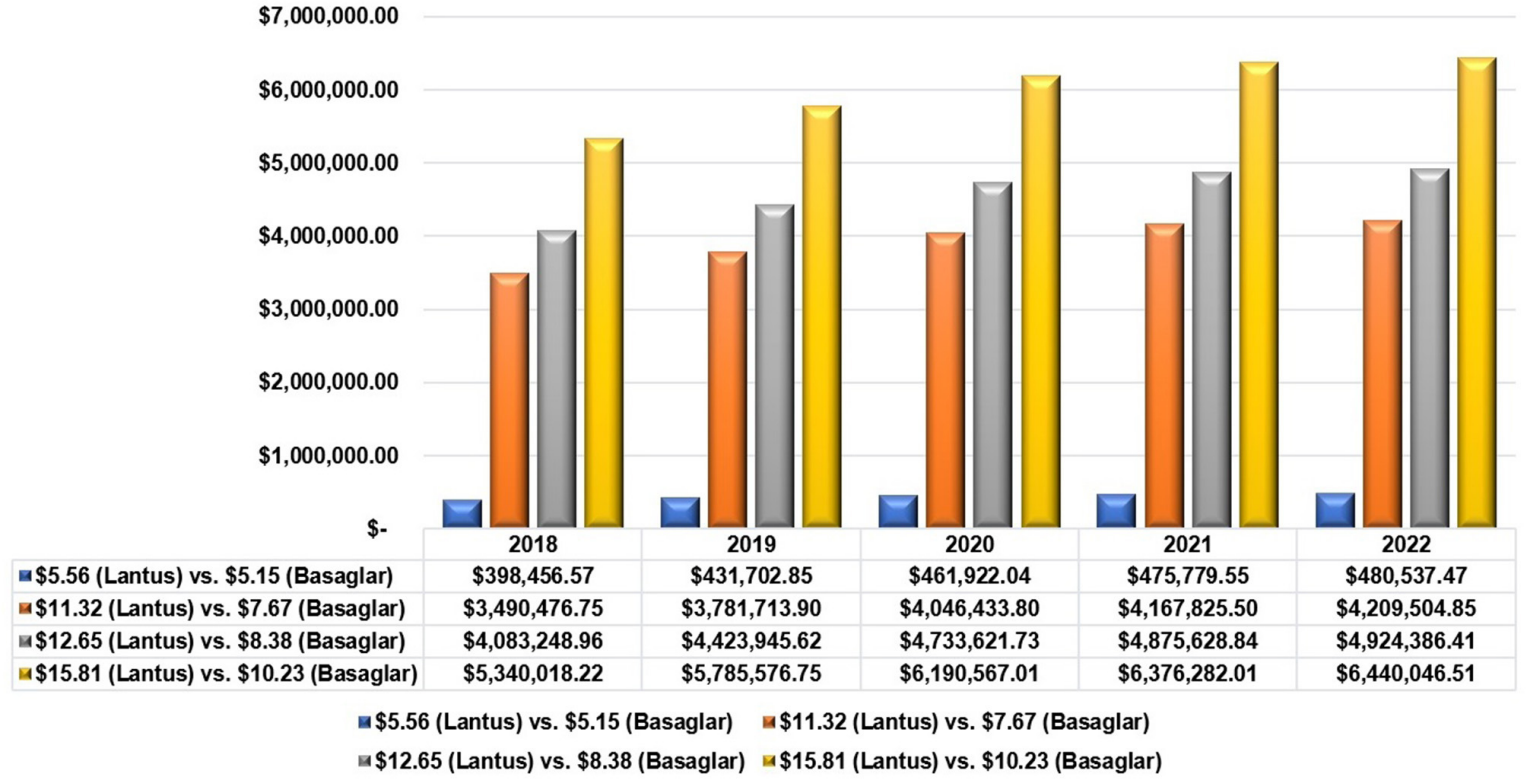
The potential cost savings due to switching from Lantus® to Basaglar® using different prices.

**Figure 2:**
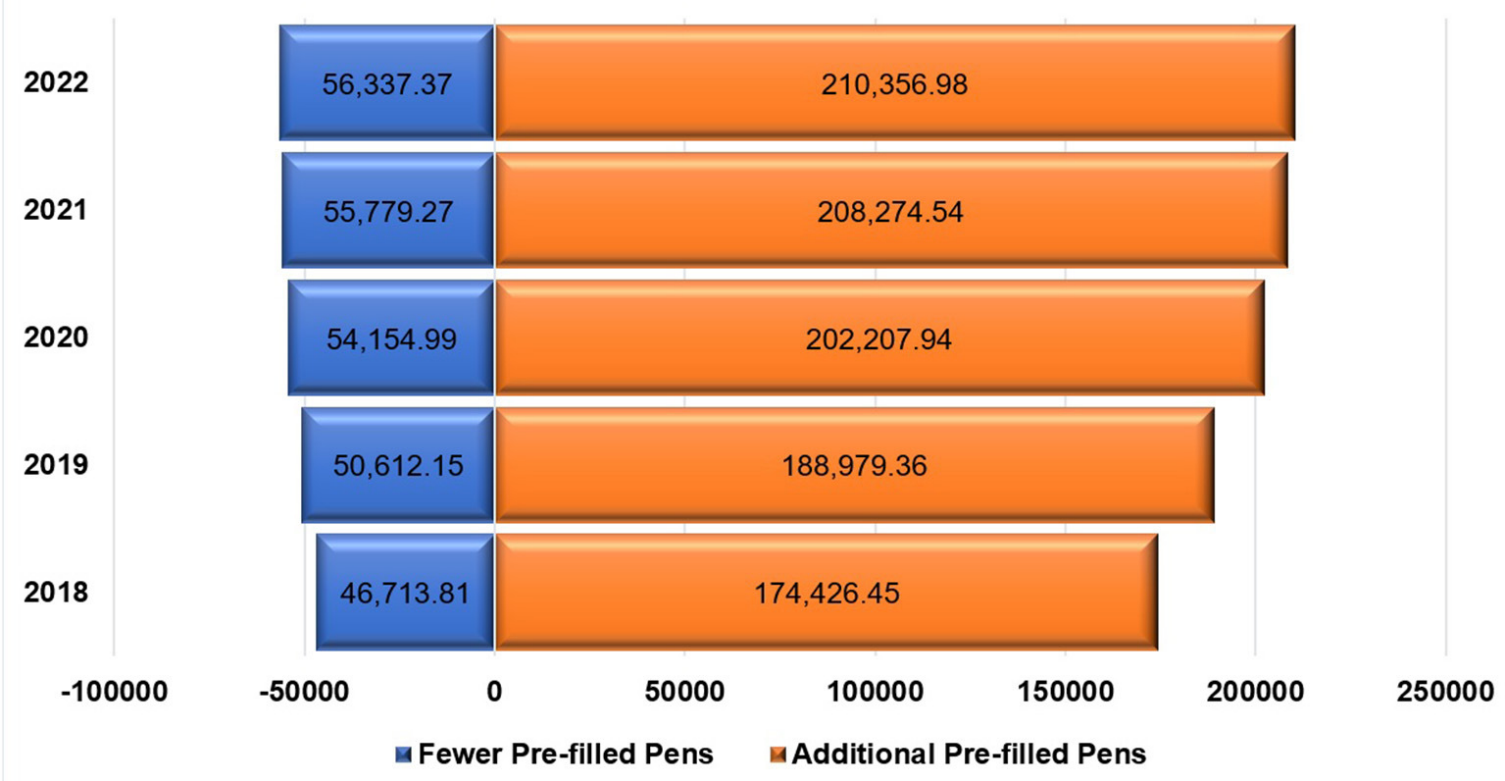
Tornado chart for the number of pre-filled pens of Basaglar^®^ needed to replace Lantus^®^ for the years 2018 to 2022 based on the 95% confidence intervals (95% CI) of mean difference in number of total daily insulin units.

**Figure 3:**
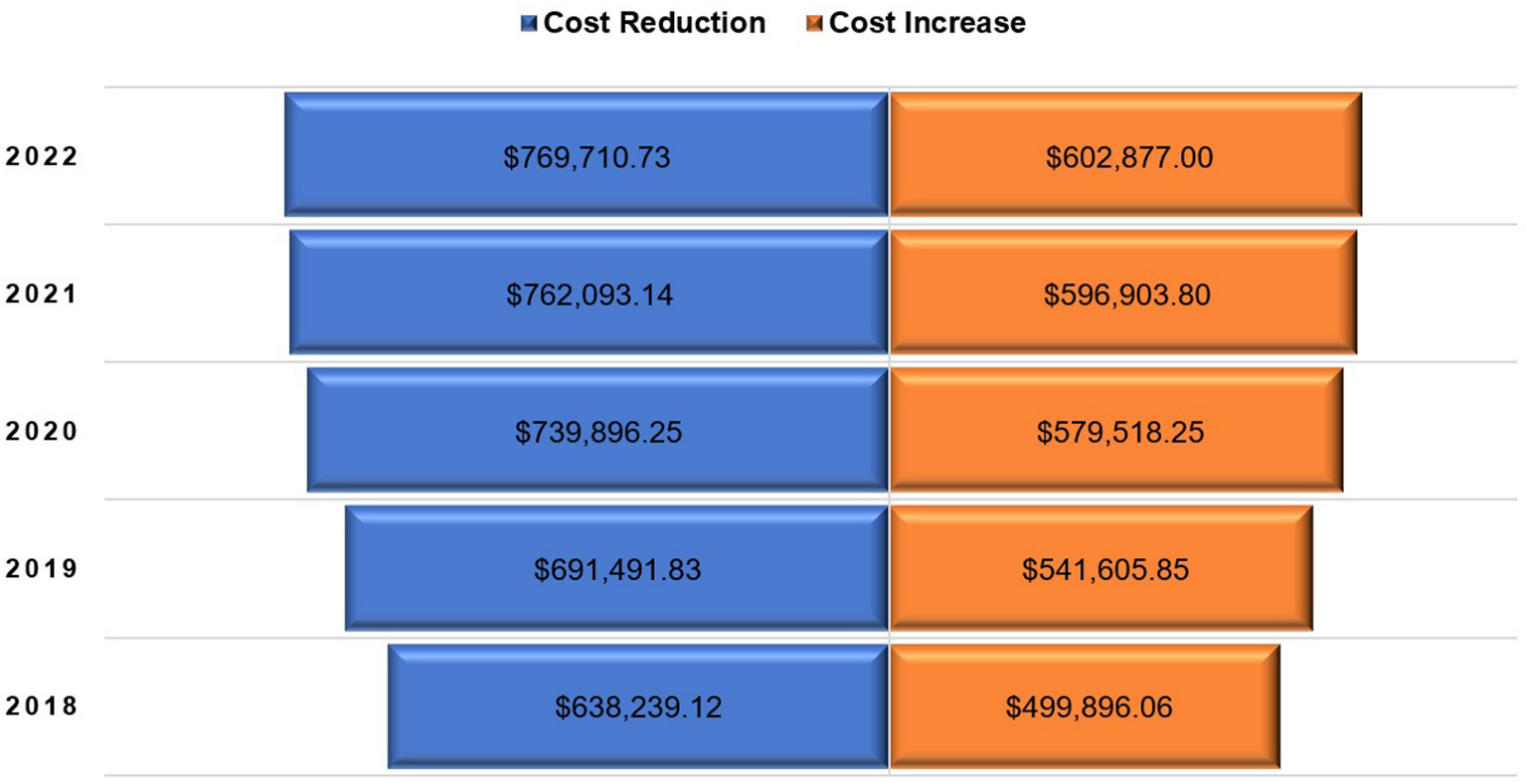
Tornado chart of potential cost reduction or increase due to complete switching from Lantus^®^ to Basaglar^®^ for the years 2018 to 2022 based on the 95% confidence intervals (95% CI) of mean difference in number of total daily insulin units.

Table 3 shows the procured quantities of Lantus^®^ and their costs for different selected prices per unit (pre-filled pen) based on expert opinions; while Table 4 shows the costs of insulin glargine if the same procured quantities of Lantus^®^ were replaced with Basaglar^®^ using different selected prices based on expert opinions as well. Figure 1 shows the expected cost savings for the years 2018 to 2022 if Lantus^®^ was switched to Basaglar^®^ using a conversion ratio of 1:1 units at different prices. However, the procured quantities can vary depending on the conversion ratio which ranges from 1:0.95 to 1:1.18 insulin units based on the 95% confidence limits for the mean difference in the total daily insulin units. These variable quantities are shown in Figure 2. Assuming the cost per unit for Lantus^®^ and Basaglar^®^ are $5.56 (SAR 20.85) and $5.15 (SAR 19.31), respectively, the potential cost reduction or increase based on the aforementioned conversion ratios for the years 2018 to 2022 are shown in Figure 3.

**Table 3:** The quantities ordered (e.g., pre-filled pens) of Lantus^®^ for a large public healthcare system in Saudi Arabia for the years 2018–2022 and their costs using different acquisition prices based on expert opinions.

**Year**	**Quantity**	**Total cost at different actual acquisition prices (AAPs) per Pre-filled pen of Lantus^®^**			
		**With an AAP of** **$5.56**	**With an AAP of** **$11.32**	**With an AAP of** **$12.65**	**With an AAP of** **$15.81**
2018	956,295	$5,319,550.64	$10,825,259.40	$12,095,726.00	$15,119,664.67
2019	1,036,086	$5,763,401.40	$11,728,493.52	$13,104,964.85	$16,381,213.84
2020	1,108,612	$6,166,839.39	$12,549,487.84	$14,022,312.14	$17,527,898.49
2021	1,141,870	$6,351,842.57	$12,925,968.40	$14,442,976.95	$18,053,729.75
2022	1,153,289	$6,415,362.66	$13,055,231.48	$14,587,410.52	$18,234,271.79

**Table 4:** The costs of Basaglar^®^ based on the procured Lantus^®^ quantities (e.g., pre-filled pens) for the years 2018–2022 ordered for a large public healthcare system in Saudi Arabia using different acquisition prices based on expert opinions.

**Year**	**Quantity**	**Total cost at different actual acquisition prices (AAPs) per pre-filled pen of Basaglar^®^**			
		**With an AAP of $5.15**	**With an AAP of $7.67**	**With an AAP of $8.38**	**With an AAP of $10.23**
2018	956,295	$4,921,094.07	$7,334,782.65	$8,012,477.04	$9,779,646.45
2019	1,036,086	$5,331,698.56	$7,946,779.62	$8,681,019.23	$10,595,637.09
2020	1,108,612	$5,704,917.35	$8,503,054.04	$9,288,690.41	$11,337,331.48
2021	1,141,870	$5,876,063.02	$8,758,142.90	$9,567,348.11	$11,677,447.74
2022	1,153,289	$5,934,825.19	$8,845,726.63	$9,663,024.10	$11,794,225.29

## Discussion

This study provides an interesting insight into the potential financial impacts if a decision to switch all insulin glargine from Lantus^®^ to Basaglar^®^ in a large military–affiliated healthcare system was taken. In Saudi Arabia, all public hospitals including those affiliated with military institutions have to procure their needs of medicines and medical supplies through a centralized procurement. Therefore, the national unified procurement company for medical supplies (NUPCO) was established back in 2009 and is believed to be the largest procurement company in the Middle East responsible for procuring, warehousing, distribution, and re-exporting of pharmaceuticals, medical equipment, and supplies on behalf of all public hospitals and healthcare facilities (31). The main aim of the centralized procurement is to cut costs and reduce waste in the public healthcare sector to improve the public health sector spending efficiency (32). Basaglar^®^, which is a biosimilar insulin glargine of the reference biologic (Lantus^®^), has shown comparable safety and efficacy profiles to Lantus^®^ among patients with type 1 and type 2 diabetes in different industry–sponsored randomized clinical trials (24, 25). Moreover, a recently published study that examined the interchangeability between Basaglar^®^ and Lantus^®^ in real–world settings found no difference in the glycemic outcomes among the 225 adult patients who were retrospectively recruited from five clinics affiliated with three different healthcare systems in the United States (27). Similar findings were observed in this study since no difference in the A1C levels was found between the baseline (e.g., before Basaglar^®^ treatment) and after 12 months of follow–up. However, there were on average about 1.9 incremental daily insulin units among this study's sample even though this difference was not statistically significant.

The use of biosimilar insulin can result in immense cost savings depending on the acquisition prices of both biosimilar insulin and the biological reference products, which is Lantus^®^ in this case (23). However, due to the uncertainty about the conversion ratio between the different insulin glargine products, such as Lantus^®^ and Basaglar^®^, and the variation in the acquisition prices of biosimilar insulins in comparison to the reference biologic products, the potential cost savings are variable (19). Although substituting generics for brand medications has resulted in significant cost savings, similar cost savings were not always realized with switching from reference biologics to biosimilar formulations. For example, Humira^®^ (adalimumab) has seen its price reduced by more than 80% since its patent expiry and the approval of several biosimilars of adalimumab (33). The inconsistent cost savings when switching from original reference biologics to biosimilars can be attributable to a myriad of factors, such as the higher cost of manufacturing biologics in comparison to small molecules, the regulatory requirements of proving bio-similarity with reference biologic products, and clinical data to support the safety and efficacy of new biosimilars (34). Nonetheless, the competition between biosimilar insulins and their reference biologics can result in significant cost reduction even if their prices are slightly lower (35).

Based on the findings of this study, switching from Lantus^®^ to Basaglar^®^ could have resulted in cost savings that would range from approximately 1.77 to 23.7 million United States Dollars (USD) for the years 2018 to 2021, and the potential cost avoidance for the year of 2022 could range from about 480 thousand USD to 6.4 million USD depending on the prices of the both Basaglar^®^ and Lantus^®^ and assuming a 1:1 conversion ratio. However, these savings might not be realized if patients with DM needed higher dosages of insulin and if the price difference between Basaglar^®^ and Lantus^®^ is smaller than 15%. Although the price difference between each prefilled pen of Basaglar^®^ and Lantus^®^ is 35.32% ($10.23 vs. $15.81) based on the SFDA public prices (36), this difference in the unit prices (prefilled pen) was not observed in the centralized tender prices based on the expert opinions. The lowest offered prices per a prefilled pen of insulin glargine for the public tenders were 5.56 USD and 5.15 USD for Lantus^®^ and Basaglar^®^, respectively, which is only 7.37% cheaper. Usually, a price reduction in the range of 20 to 40% for biosimilars should be provided to justify switching from a biological reference product to a biosimilar (19). Therefore, a reduction in the price of Basaglar^®^ by 20% to 40% should be provided for public tenders to make the switching financially worthwhile. By discounting the prices of biosimilars in general and biosimilar insulins in particular in comparison to their original reference biologics, the rates of biosimilar adoption and inclusion in different public healthcare systems' drug formularies will increase leading to more efficient utilization of healthcare resources in the kingdom, and better accessibility to medicine for the public as advocated by the Saudi economic vision 2030 (37).

Although this is the first study to the best of our knowledge that examined the impact of switching from insulin Lantus^®^ to Basaglar^®^ on the glycemic outcomes and budget from the perspective of public healthcare payers in Saudi Arabia using real-world data, multiple limitations must be acknowledged. First, the study included only 100 patients which limit the generalizability of the results and increases the likelihood of selection bias. In addition, information bias cannot be ruled out since the data were retrieved from the EMRs. Moreover, the budget impact was estimated based on the procured quantities rather than the number of patients with DM who are treated with insulin glargine due to the lack of data about the real number of patients with DM in these healthcare institutions. Additionally, the selected prices upon which budget impact was examined were based on expert opinions.

## Conclusion

The biosimilar insulin glargine (Basaglar^®^) has shown similar glycemic outcomes to its biological reference product (Lantus^®^) with insignificant difference in the mean total daily insulin dose. However, these slight differences in the total daily insulin dose could offset any cost savings attributable to the lower acquisition price of Basaglar^®^ in light of the meager difference between the public tender prices of the two products (Basaglar^®^ and Lantus^®^). Therefore, an additional discount in the public tender price of Basaglar^®^ in the range of 20% to 40% should be provided to justify the switching from Lantus^®^ to Basaglar^®^. Future studies should further examine the interchangeability between the two insulin glargine products using a more robust research designs and among a larger sample of patients with DM.

## Data Availability Statement

The raw data supporting the conclusions of this article will be made available by the authors, without undue reservation.

## Ethics Statement

The study was approved by the Ethics Committee of the King Abdullah International Medical Research Center, Riyadh, KSA (approval no. RC20/608/R). Written informed consent for participation was not required for this study in accordance with the national legislation and the institutional requirements.

## Author Contributions

YA, HA, and OB: concept and design. YA, HA, OB, MoA, AA, SA, and MaA: acquisition, analysis, and interpretation of data. YA and OB: drafting of the manuscript and statistical analyses. SA, HA, MoA, HA-A, MaA, and AA: administrative, technical, and material support. All authors have read, agreed to the published version of the manuscript, and critical revision of the manuscript for important intellectual content.

## Funding

This research received financial support from the Researchers Supporting Project number (RSP-2021/16), King Saud University, Riyadh, Saudi Arabia.

## Conflict of Interest

MaA is an employee of the National Unified Procurement Company (NUPCO). The remaining authors declare that the research was conducted in the absence of any commercial or financial relationships that could be construed as a potential conflict of interest.

## Publisher's Note

All claims expressed in this article are solely those of the authors and do not necessarily represent those of their affiliated organizations, or those of the publisher, the editors and the reviewers. Any product that may be evaluated in this article, or claim that may be made by its manufacturer, is not guaranteed or endorsed by the publisher.
